# Global Health Security amid COVID-19: Tanzanian government’s response to the COVID-19 Pandemic

**DOI:** 10.1186/s12889-023-14991-7

**Published:** 2023-01-31

**Authors:** Nyaso Malilo Hamisi, Baozhen Dai, Masud Ibrahim

**Affiliations:** 1 School of Management Jiangsu University, Zhenjiang, China; 2 School of Public Health, Southeast University, Nanjing, China; 3 Department of Management Studies, AAMUSTED, Kumasi, Ghana

**Keywords:** Global Health Safety, Global Health Vaccination, COVID-19 pandemic, Global Health Security, Tanzania Health system

## Abstract

Several stakeholders assumed different responsibilities for global health security and safety during the COVID-19 pandemic. This study aims to highlight how the Tanzanian government, in collaboration with the international government, non-governmental organizations (NGOs), donor agencies, and stakeholders responded to the pandemic to improve health security and community well-being. This article analyzed secondary data sources from the World Health Organization’s (WHO) country report and published reports from Tanzania’s government to evaluate vaccine availability and health security. Findings from the data gathered indicate that the initial response from the Tanzanian government concerning the fight against the COVID-19 pandemic was rather abysmal due to the posture of the late President John Pombe Magufuli who at first downplayed the severity and seriousness of the COVID-19 pandemic. However, with the swearing-in of the new President, Samia Suluhu, there was a new approach and strategy instituted to deal with the pandemic which has resulted in the country making headway in containing the pandemic. Data gathered thus, indicate that, as of 11^th^ February 2022, the total number of fully vaccinated individuals in the country as of 12th April 2022 stood at 3,435,513 from the total number of 2,205,815 reported on 11^th^ February 2022. This study thus, concludes that there is a need for a strong stakeholder engagement with high-level political, community, and religious leaders and increased access to COVID-19 vaccination as well as a mass campaign to scale up vaccination activities with adequate resource mobilization and plan.

## Introduction

Global health security is described as the proactive and reactive measures needed to reduce the risk and impact of acute public health events that threaten people’s health across geographical regions and international borders [1]. However, health and security are linked to various disciplines and their use in various contexts (individual, national, and global) for various goals. Health security, for example, refers to all aspects of public health that protect the critical core of human life at the individual level [2, 3]. The term ‘health security is commonly used at the national and international levels to refer to safeguarding people against public health hazards such as infectious illnesses and bioterrorism, which constitute a threat to national and international peace and stability [4].

Challenges from infectious diseases, whether natural, unintentional, or deliberate, pose serious threats to global health security and the economy. The novel coronavirus (COVID-19) pandemic serves as a stark reminder that global health security must be prioritized. Since the outbreak of the COVID-19 pandemic in March 2020, there have been several studies on government and non-governmental responses to global health security and global vaccination programmes [5–9]. Roberts and Kelman [7] highlight that the securing and rolling out of vaccine programmes reveals the distinct governance, political, and geopolitical realities of different-sized countries within global health security frameworks. While government, NGOs, and businesses’ responses to COVID-19 have been analyzed for many developed and developing economies, East African stakeholders’ responses remain unexplored, hence, the contribution of this article.

Vaccination was the most critical means to end the pandemic, hence, equitable access to safe and effective vaccines was considered by global health experts and governments as crucial, and no country must not be left behind. Despite the importance of vaccination, rates of vaccination in African countries remain low. According to figures from [10] as of July 2022, only 24.4 percent of the population had received at least one dose, compared with the global average of 69.0 percent. Against this backdrop, it is critical to evaluate the conditions and characteristics of African health security amid COVID-19. Therefore, research concerning vaccine availability, vaccine cost, vaccine acceptance, or sentiment requires further investigation.

Tanzania like other African governments has responded to the global call for health security and well-being. Through the practice of Global Health Diplomacy, the country collaborates with some stakeholders in the health sector, as well as foreign and other global stakeholders. This study therefore seeks to examine the Tanzanian government’s response to the COVID 19 pandemic as well as find out how the government is collaborating with other stakeholders to in responding to the pandemic to improve global health security and community well-being. The Global Health Security Agenda (GHSA) is a multisectoral, multinational endeavor including almost 70 countries, as well as the commercial sector, civil society, and international organizations. Its goal is to help countries improve their ability to prevent, identify, and respond to infectious disease risks [11]. The International Health Regulations (IHR) [12] establish a comprehensive legal framework that explicates countries’ obligations and rights in handling public health incidents and emergencies that have the potential to cross boundaries. The IHR is a piece of international law that has legal force in 196 countries including the 194 WHO Member States.

The United State Agency for International Development (USAID) collaborates with the Tanzanian government to prevent, identify, and respond to new infectious illnesses as part of the Global Health Security Agenda (a collaboration of over 60 countries focused on minimizing pandemic threats). Recognizing the risk of animal-borne diseases spreading to humans, USAID funds Tanzania’s “One Health” coordination desk, which coordinates the preparedness and response across sectors such as public health, veterinary care, law enforcement, and the environment/wildlife. The consequences of COVID-19, such as the overburdening of entire health systems, the loss of life, and the resulting global economic disruptions, highlight the importance of every country prioritizing investment in health security and building capacity to combat infectious disease threats.

## Pandemic Diseases and Control

Diseases such as the COVID-19 virus and Ebola, are surfacing at unprecedented rates, wreaking havoc on people’s health and wreaking economic and social havoc. The chances for rapid international diffusion are greatly enhanced by cross-border mobility. When the WHO Director-General designated COVID-19 a Public Health Emergency on January 30, 2020, the WHO continued to work with African countries to improve Covid-19 surveillance, with the primary goal of quickly locating, testing, isolating, and identifying suspected cases, as well as quarantining persons who have had close contact with confirmed cases.

GHSA’s involvement in tackling these difficulties at the country level, as well as its efforts to incorporate lessons learned from each event into its work, has improved the world’s preparedness. In today’s globalized world, health challenges cannot be solved by the health sector alone. It requires the cooperation and engagement of both state actors and non-state actors to prepare an adequate response to tackle the challenges.

Health security has been widely discussed in the global context to ensure the well-being and protection of the community against pandemics and infectious diseases. Fostering partnerships across sectors for public health, engaging diplomats in global health affairs, and advocating healthy causes are part of the practice to achieve social justice, economic development, and national security. Therefore, this study is going to explore the role played in strengthening national security through global health diplomacy and examining Tanzania’s response to the COVID-19 pandemic to minimize societal disruptions and alleviate community transmissions.

Furthermore, a detailed presentation on the strategies and roles played to strengthen the global health security involved in this research may serve as a tool for further studies and future discussion on leadership practice in responding to communicable diseases. However, the highlight of the GHSA (Detecting, Prevention, Respond) discussed will contribute and make a significant contribution to tackling the health challenges that countries are facing today.

## Stakeholder Engagement and Global Health Security

Since the outbreak of Covid-19 (2019) epicenter in Hubei province in the People’s Republic of China, the WHO has worked towards ensuring the prevention and containment of the Virus. Millions of passengers traveling each year increased the chance of rapid international spread. The WHO has been working hand in hand with different countries to provide technical support to ensure the situation is controlled. Nevertheless, several scientists and researchers also worked hard to provide their support by investigating the COVID-19 epidemic history and mostly on the outcomes and challenges regarding global health security. However, this research paper is innovative because it seeks to identify the leadership practices during pandemics in response to the global health security agenda. Building capacity, managing capabilities, and adapting to change are components of stakeholder engagement and management [13]. The paper is also innovative because it highlights measures that have been implemented to strengthen health systems and ensure effective outcomes. The study also provides the importance of addressing health challenges by involving other stakeholders rather than government alone.

## Methodology

A qualitative research method was adopted to examine the engagement of Tanzania in improving global health security and the well-being of the people during the COVID-19 pandemic. In this review, secondary materials were used to gather data for the study. About 20 documents were reviewed obtained from the Tanzania government’s publications, the official records of both WHO and UN General Assembly meetings, National Health Databases, Tanzania’s Ministry of Health Website, UN database as well as WHO previous reports. To find relevant articles for this review, Web of Science, PubMed, UN/WHO databases, and Google Scholar search engines were used to find published articles during the period 2020–2022. Data were extracted from each eligible study. Keywords with a common term related to the topic such as ‘Health Diplomacy’, ‘Global Health Security’, ‘COVID-19’, ‘Global Health Diplomacy’, etc., were used for the search. Any documents published in any other language apart from English were not included. A total of 18 academic articles and 13 reports were used for the study’s review. A content analysis of published materials was done to achieve the objective of the study. Content analysis according to scholars is used to reduce and interpret qualitative data to identify core meanings and consistencies [14].

## Results of the Study

### Tanzania’s leadership practice and approaches during the COVID-19 Pandemic

When the first imported case of COVID-19 was reported in the WHO African Region in February 2020, all nations in the regions responded with varying degrees of adequacy, with coordinated support from the regional office and all partners [15]. Community fortitude and perseverance, African governments’ strong leadership, and partners’ support have all played critical roles in limiting the spread of COVID-19 throughout Africa. Different measures were in response to WHO’s advice so to defeat the COVID-19 virus and ensure the well-being of the African Region. Many countries closed down schools and other activities including religious and social activities. Many locked down the country, however, Tanzania’s late President John Pombe Magufuli refused to lock down the country as neighbors. He spoke out about his doubts about imported masks and testing kits.

Tanzania reported its first COVID-19 case on March 16, 2020, involving a returning national who re-entered the country through Kilimanjaro International Airport. In mid-April, the Ministry of Health announced the transmission of COVID-19. More cases were confirmed and deaths were recorded after a month. Four Hundred and Eight (408) confirmed cases were reported on May 2, 2020, and new preventive measures were considered to combat the virus.

During the government of late President John Pombe Magufuli, face masks were required in densely populated areas such as markets, public transportation, and health facilities, as well as the prohibition of all large public gatherings, the avoidance of unnecessary gatherings, the limitation of the number of people attending burials, the use of sanitizer, the installation of hand-washing facilities in public places and households, the earmarking of isolation and quarantine centers, and the expansion of diagnostic points of service. This was to prevent the spread of the COVID-19 virus.

However, the late President Hon. John Pombe Magufuli, downplayed the seriousness and severity of the virus. Notably, he never shut down the churches and mosques and allowed religious services to continue, nor did he impose a curfew. People were strongly encouraged to continue with their income-generating activities. In addition, the government of President Magufuli boycotted the use of vaccines and rather recommended the use of traditional medicines which delayed the implementation of the WHO recommendations for the use of vaccines to combat the deadly COVID-19 virus.

Furthermore, public data on the number of people suffering from the COVID-19 virus was not released since the end of April. His message was different and insisted that people should regard the COVID-19 virus just like any other disease they suffer and learn to live with the virus. In that regard, the then President never closed the border and allowed tourists to visit without any restrictions.

Magufuli, who refused to accept vaccines again urged people to pray in order to battle the Corona Virus. The Vice President, Samia Suluhu, announced the death of President Magufuli on March 17, 2021, making her Tanzania’s first female president. As Vice President since 2015, she was then recognized as Tanzania’s President and was asked to serve out the remainder of Magufuli’s five-year term as the new President of Tanzania.

The new President immediately after assuming the leadership of the country has had to deal with several difficulties. The country was still dealing with Covid-19, which appeared to be the most severe wave ever. One of the only sources of information on the disease’s prevalence in the country at the moment—indicated that the case numbers were declining, but prominent persons continued to get COVID-19 like symptoms. International health authorities had to put renewed pressure on the government to start publishing statistics and accepting foreign aid, such as vaccines. While Tanzania had fared better than many other countries in dealing with the COVID-19 related economic shocks, growth had lagged below its potential and continued to do so till the government began promoting science-based public health approaches.

President Samia Suluhu presented different approaches and started working towards halting the spread of the virus. She promised that Tanzania will continue to honor its obligations under international law, including those regarding human rights and gender. Tanzania, like others in the region, accepted the vaccines and also the President’s plea and advice for the people to be vaccinated in order to stay safe [16].

From 3 January 2020 to 6:02 pm CET, 3 February 2022, COVID-19 confirmed cases were 32,920 with 778 deaths, reported to WHO. As of 28 December 2021, a total of 2,431,769 vaccine doses were administered [16]. Tanzania supports the spirit of working collaboratively with international and local agencies to fight against the COVID-19 pandemic. Awareness of taking sensible precautions and preventive steps to reduce the risk of infections was created. More figures on COVID-19 cases were released on January 3^rd^, 2020. As of 3^rd^ October 2022, the total confirmed COVID-19 cases reported stood at 39,513 with 845 deaths reported to WHO. Also, with regards to the vaccination drive, as of September 2022, a total of 25,039,939 vaccines have been administered in Tanzania.

Under Magufuli’s government, Herbal steam, Natural remedies, and exercise were the main interventions introduced by the health ministry to fight against the COVID-19 pandemic in Tanzania. However, after the demise of the President and with the ascension of the new President, a change of policy in the fight against COVID-19 was introduced by the new President. The government through the Minister of Health, Dorothy Gwajima appealed to people to be vaccinated as the Government planned to at least get about 60% of the population vaccinated. Between the period of July 2021 and July 2022, Tanzania has received a total number of more than 2.9 million Jansen vaccines, more than 5.6 million of Sinopharm and 4.1 million of Pfizer, more than 376,320 doses of Moderna and over 1 million Sinovac vaccines (https://tz.usembassy.gov, http://www.statista.com/statistics/1258567/total-number-of-covid-19-accinatio-doses-in-tanzania/).

Through the COVAX initiative, Tanzania received the first batch of over one million Covid-19 vaccines donated by the United States of America and officially launched the campaign in July 2021 [17]. As part of the USA Government’s efforts to end the pandemic globally, it has so far donated over 4.5 million COVID-19 vaccine doses to Tanzania which includes over 3.5 million doses of the Pfizer BioNTech that were dispatched between November 2021 to January 2022 (http://www.statista.com/statistics/1258567/total-number-of-covid-19-accinatio-doses-in-tanzania/) [17]. Between November 2021 and July 2022, the Chinese government gave more than 5.61 million Covid-19 vaccinations. In order to get 60% of the populace immunized by the end of June 2022, the United Republic of Tanzania’s ministry of health continues to work with the second Intra Action Review (IAR) of the country, which was conducted after the first review of the response took place in October 2021 as a result of the introduction of additional vaccines, was completed. Other development partners, including WHO, the British Council, UNICEF, USAID, and the Centers for Disease Control and Prevention (CDC), were given technical support in response to the COVID-19 pandemic (Moderna, Pfizer, and Sinopharm). Since then, Tanzania has increased its efforts to immunize against COVID-19, expanding outreach efforts and revising its National Vaccine Development Plan (NVDP) Worldwide Vax makes ensuring that rural populations have access to vaccinations, data, and analytics and strengthens the capacity of health workers to assist vaccination efforts in order to achieve the global objective of immunizing 70% of the world’s population against Covid-19 in 2022. The International Monetary Fund (IMF) awarded US$ 372.4 million in emergency financial assistance to deal with the COVID-19 Pandemic in November 2021 under the Rapid Credit Facility (RCF).

As of June 19, 2022, over 8.8 million doses of COVID-19 vaccines had been administered (http://www.statista.com/statistics/1258567/total-number-of-covid-19-accinatio-doses-in-tanzania/). In collaboration with other initiatives, Tanzania proceeded to emphasize the importance of vaccination in the communities which helped to increase patronage and saw the number of people fully vaccinated exceeded 18 million as reported by the Ministry of Health of Tanzania. About 13.7 million people received the Johnson & Johnson (J&J) vaccines whiles over 2.5 million people received the Sinopharm vaccine, and 1.3 million received the Pfizer vaccine. Also, 293,293 doses of Sinovac and 98,415 doses of Moderna [18] were administered.

### Tanzania’s engagement with other stakeholders in Global Health Diplomacy

The USAID has committed more than $5.75 million ($3.4 million in new resources and $2.4 million in redirected existing funds) to help Tanzania respond to the COVID-19 pandemic [19]. The intervention focused on health risk communications, laboratory capacity for optimal diagnostics, public health messaging, water and sanitation, infection prevention and control, and virtual telecommunications (see Table 1).

**Table 1:** Different projects implemented by USAID in Tanzania in response to COVID-19

Projects Implemented	Activities
Boresha Afya/Improve Health	● Supports local health institutions in the implementation of COVID-19 strategic approaches. ● Strengthens the capacity of healthcare workers to effectively manage the patient flow for early detection, isolation, and provision of high-quality care while adhering to infection prevention and control guidelines. ● Collaborates with community health workers to provide community-facility referrals as well as implement social mobilization activities through the dissemination of social behavior and communication materials for prevention and response to COVID-19.
Promoting Tanzania’s Environment, Conservation and Tourism (PROTECT)	● PROTECT is developing online training materials to help investigators, prosecutors, and other law enforcement agencies respond appropriately to the short and long-term impacts of Covid-19 and other zoonotic diseases.
Medicines, Technologies, and Pharmaceutical Services (MTaPS)	● COVID-19 training to health care workers, volunteers, and triage units through an e-learning platform
Data for Development(D4D)	● Assessing how COVID-19 is impacting youth and USAID programming in Tanzania ● Conduct interviews, and provide recommendations to help USAID/Tanzania adjust activities to mitigate the immediate and secondary impacts of Covid-19.
Boresha Habari (“Improve the News”)	● Supports small grant assistance to community radio stations to create and air COVID-19 messages and to provide stipends for journalists.
Data-driven advocacy	● Provides technical assistance, expert advice, and legal and practical guidance to senior political and technical staff within key Government of Tanzania institutions and bodies related to the creation of vision, policy, and oversight of the enforcement of Covid-19 public health and safety related measures
Waache Wasome (“Let them Read”)	● Providing school and Covid-19 prevention materials, procuring media equipment, and public broadcasting
Tulonge Afya (“Let’s Talk About Health”)	• Provides platforms that amplify social behavior change communications in Tanzania to better inform, motivate and empower youth to adopt healthy behaviors that will improve their overall well-being.

Source: Prepared by the authors based on the available data (SADC Report, 2021)

The money from the United States builds on the United States’ long-term support for Tanzania. Despite this, the US has invested more than $7.5 billion in Tanzania over the last 20 years, with about $4.9 billion going to health [20]. For more than a half-century, the US government has been the leading supporter of global health security and humanitarian aid.

As part of an All-of-America response, USAID, the State Department, the Centers for Disease Control and Prevention, the Department of Defense, and others contributed to global health systems, security, humanitarian assistance, and economic stability.

The Tanzanian Government in collaboration with other international organizations and non-governmental organizations within the country entered into a public/private partnership in an attempt to support the global anti-pandemic efforts through various projects (see Table 2). For instance, the government partnered with some global organizations like the United Nations Children’s Education Fund (UNICEF), and Médecins Sans Frontiers (MSF) to train aid workers in some communities like Nyarugusu and Mtendeli Camp to ensure the promotion of appropriate Infant and Young Child Feeding (IYCF) practices during the COVID-19 Pandemic.

**Table 2:** Public-Private Partnerships in response to the COVID-19 pandemic in Tanzania

ORGANISATIONS	AREAS IMPLEMENTED	ACTIVITIES
UNICEF together with Médecins Sans Frontiers (MSF) and Tanzania Red Cross Society (TRCS)	Nyarugusu and Mtendeli Camp	A total of 50 Health information teams (HITs) and 25 Community Nutrition Volunteers (CNV) were trained to ensure the promotion of appropriate Infant and Young Child Feeding (IYCF) Practices during the Covid-19 Pandemic.
UNICEF	Mbeya Region, Iringa Region, Njombe and Songwe	During COVID-19, to ensure the continuity of essential nutrition services in health facilities. 3,949 children 6-59 months were admitted and treated, out of which 2,595 (80 percent) recovered
UNICEF Collaborates with Tanzania Food and Nutrition Centre	Tanzania Mainland and Zanzibar	In various aspects of COVID-19, 1,198 CHW (Community Health Workers) were trained in response including nutrition
UNICEF		To ensure health services sustainability in the refugee camps during the COVID-19 pandemic Maintenance of essential services for adolescents, children, and pregnant women through the provision of medicines, 57,080 children and women benefitted from equipment and supplies
The national NGO, Benjamin Mkapa Foundation (bmf) in collaboration with UNICEF	Tanzania mainland and Zanzibar	A total of 565 Community Health Workers (chws) were provided mobile smartphones that are installed with a purpose-designed mobile health application to ensure the timely collection of basic data reported by the community. 1.4 million households were reached and over 5 million people with covid-19 prevention messages and educated on other essential services through 620 community health workers (chws)
UNICEF in partnership with the Presidents’ Office-Regional Administration and Local Government (PORALG), the ministry of health, community development, gender, elderly and children (MoHCDGEC), BMF, and Local Government Authorities (LGAS)	Tanzania mainland Zanzibar	42 national tots (19 in Zanzibar and 23 in Tanzania mainland) were trained on risk communication for highly infectious diseases including covid-19 and Community-Based Surveillance (CBS).
	Dar es Salaam Region	57% of the population was reached with messages on covid-19 prevention and continual utilization of essential services in the region.
UNICEF	Tanzania Mainland and Zanzibar	Supplies of 45,564 masks; 38,458 aprons; 46,030 gloves; 4,300 safety boots; 93,356 respirators (n95); 36,446 safety glasses; 1,656 face shields;34,213 hand sanitizers; 880 sharp containers;893 waste bins; 1,546 thermometers; 550 pulse Oximeter; 62,884 gowns; 2,540 body bags; 217 oxygen concentrators; 370 flow splitters for oxygen concentrators and 9,730 nasal prongs.
Management And Development for Health (MDH) in collaboration with UNICEF	Dar es Salaam Region	Supplies of 10,000 face masks to some health facilities. 3,857 children, adolescents, and pregnant women living with HIV were provided with 20,000 reusable masks and over 18,000 soap bars.

Source: UNICEF, Tanzania Humanitarian Situation Report No. 4 (January-December 2020)

UNICEF also worked in the following regions in Tanzania Mbeya, Iringa, Njombe, and Songwe to ensure the continuity of essential nutrition services in health facilities. About 3,949 children aged 6-59 months were admitted and treated, out of which 2,595 (80 percent) recovered. UNICEF also collaborated with the Tanzania Food and Nutrition Centre in the Tanzania Mainland to train over 1,198 Community Health Workers in various aspects of COVID-19 including nutrition.

### Tanzania’s approaches to strengthening health security and wellbeing

Infectious disease challenges exist everywhere, whether they are unintentional, natural, or intentional. The COVID-19 epidemic serves as a stark reminder that global health security must be addressed. The Global Health Security Agency, which was established in 2014 and was renewed in 2018 for a second five-year phase, is a multisectoral, multinational endeavor involving almost 70 countries, the commercial sector, civil society, and international organizations. Tanzania is included in the first phase. Its goal is to help countries improve their ability to prevent, identify, and respond to infectious disease risks. Capabilities at the country level are being strengthened in order to avoid, identify, and respond to public health emergencies.

Nonetheless, according to recent statistics, Tanzania is ranked 124th in the Global Health Security Index (GHSI), which assesses 195 countries’ preparedness for epidemics and pandemics (see Table 3). Tanzania was assessed for its IHR country core capacities in February 2016 using the Joint External Evaluation Tool developed by WHO in partnership with partners, in particular the Global Health Security Agenda [21].

**Table 3:** Key Components of Global Health Security

Overall Rank	124/195
Overall, By Region-Africa	18/54
CATEGORY 1	
Preventive Indicators	
Antimicrobial Resistance AMR	130/195
Zootonic Disease	90
Biosecurity	70
Dual use research and culture of responsible science	13
Immunization	101
CATEGORY 2	
Detect Indicators	
Laboratory system s strength and quality	82/195
Laboratory supply chain	58
Realtime surveillance and reporting	146
Surveillance data accessibility and transparency	160
Case-based Investigation	95
Epidemiology workforce	1
CATEGORY 3	
Respond Indicators	
Emergency preparedness & Response Planning	181/195
Exercising response plan	19
Emergency response operation	145
Linking public health and security authorities	44
Risk communication	191
Access to communication infrastructure	172
Trade and travel restriction	1

Source: Prepared by the authors based on the available statistics (GHS Index 2022)

Tanzania’s healthcare system is multifaceted and intricate. It consists of corporate, governmental, and donor stakeholders who operate at a variety of scales, including local, district, regional, and national levels. It may also be thought of as having six fundamental components, including human resources for health (HRH), finance, information, governance, supply chain, and service delivery [22]. For a nation to be able to safeguard its population when a health emergency or disaster strikes, a strong health system is very necessary. The United Republic of Tanzania’s National Action Plan for Health Security (NAPHS) (2017–2021) is the first Action Plan created utilizing a multisectoral strategy that has encompassed knowledge from multiple sectors and enhanced collaboration when addressing national health security. This National Health Security platform was created as a coordination tool to map and guarantee interaction between various sectors and other existence plans at all administrative levels in the nation.

A multi-sectoral approach was adopted by the Tanzanian government in fighting the COVID-19 pandemic. This is a collaboration among various stakeholders and sectors with a common vision and perspective to jointly achieve the desired outcome of fighting the COVID-19 scurge [23]. The Ministry of Health of Tanzania understood that adopting a multisectoral approach is important in strengthening the health security and well-being of the people. This entailed collaborating among actors at multiple levels, such as international agencies, religious institutions, communities, donors, government ministries, and civil society organizations as well as across different sectors such as education & training, health finance, and planning to fight the COVID-19 pandemic. This offered the government strength in preparing and responding timely to the COVID-19 pandemic.

#### National/ Government sectors (Inter ministerial steering committee roles) for strengthening National Health Security

The following were some of the multisectoral approaches adopted by the government in responding to the COVID-19 pandemic.

- Ministry of Health, Community development, Gender, Elderly and Children (MoHCDGEC)

This ministry acted as a leading Ministry for the whole implementation of health strategies and plans to ensure the safety of the community in response to the pandemic

- Ministry of Education, Science, and Technology (MoEST)

Due to the effect of coronavirus disease (COVID-19), MoEST in collaboration with President Office-Regional Administration and Local Government (PO-RALG) and other educational stakeholders developed The Tanzania Basic Education Sectors Response and Recovery Plan. The plan provided strategies for basic education in response to the COVID-19 pandemic which sought to protect children from contracting the virus and allow them to continue their studies at home virtually [24].

- Ministry of Finance and Planning (MoFP)

The Ministry of Finance and Planning was tasked primarily with the role of expending resources for the supply of needed equipment and tools to fight the COVID-19 pandemic. Under the Government of the United Republic of Tanzania, MoFP in collaboration with other sectors worked towards ensuring the implementation of the Tanzania Covid-19 Socioeconomic Response and Recovery Plan (TCRP).

- Ministry of Natural Resources and Tourism-

Through covid-19 capacity building, private actors in the tourism sector were engaged. A total of 3,523(90.5%) tourism business operators were trained on adherence to international guidelines and national standards of operating procedures for COVID-19. A total of 1060 tour guides were trained to cope with the COVID-19 pandemic [24].

- Ministry of Water and Irrigation

The water sector has been one of the important interventions in the fight against COVID-19. It focused on the expansion, rehabilitation, and extension of water supply in both Rural and Urban areas. Over 170 water supply projects in rural area and 46 projects in urban area was implemented to ensure access to water in the community [25].

- Ministry of Foreign Affairs and International Cooperation (MoFAIC)

Through embassies and regional organizations, the ministry’s role was to collaborate in the dissemination of information to other countries.

- Ministry of Information, Youth, Sports, and Culture

To ensure the implementation of the National action plan for Health security, the ministry has a primary role in emergency public information, warning and supporting communication and information dissemination to the public.

## Discussion

This paper illustrated Tanzania’s response to the COVID-19 pandemic. It emphasized that the initial stage of the country’s fight against the COVID-19 pandemic was a bit shambolic and chaotic due to the stance of the then-leader of the country President Magufuli. Whiles other countries were enforcing the pandemic strategies as advised by the WHO, the then President was downplaying the seriousness of the pandemic and was recommending unscientific methods to deal with the pandemic.

Again, the state refused to impose restrictions on the movement of persons and also did not close the borders as recommended by the WHO to help prevent people with the virus to enter or exit the country. This refusal by the country to lockdown could be due to the untold hardships that the lockdowns could bring on the already suffering masses who are struggling to feed themselves. Also, as reported in an earlier study, Mfinanga et al. [26] mentioned that the state officials feared that lockdowns would prevent ‘public access to health services, especially for patients with chronic conditions like tuberculosis and HIV infection’ [27]. Furthermore, as reported by Global Fund, the reasons why the state refused to lockdown the country is due to the fear of contracting coronavirus at health facilities, and limited access to transportation which undermined access to lifesaving medications for people with HIV, tuberculosis, and malaria in 24 African countries [28].

Furthermore, findings from this study revealed that to effectively combat the COVID-19 pandemic, the country had to engage with key stakeholders like the WHO and other international organizations like UNICEF, to streamline the country’s efforts towards the fight against the pandemic. Thus, adopting a multisectoral approach to fight the COVID-19 pandemic and cooperating with other international organizations played a big role in strengthening the country’s health system.

National and international stakeholders (comprising governments, NGOs, businesses, and communities) played a big role in strengthening the healthcare system of Tanzania. For most of the pandemic, Tanzania left the health and economic effects of the COVID-19 pandemic largely unattended. The local presence of the virus was denied, and the impact of the pandemic in the country was downplayed. As a result, the government did not provide sufficient resources to address the health crisis and support affected economic sectors and households. While the government did impose restrictions on movement and gatherings earlier in the pandemic, these were lifted in June 2020. The government stopped reporting COVID-19 data in May 2020 (at that time, COVID-19 cases stood at 509 and fatalities at 21) and chose not to sign up for the COVAX facility. The official response also undermined confidence in the country’s testing capacity and fomented doubts about the benefits of vaccines.

However, the new President took a new stand by opening up and being more transparent towards the pandemic, acknowledging the presence of COVID-19, and putting in measures to address the devastating effect of the pandemic. The new administration’s openness further led to an overwhelming increase in the number of vaccinations recorded by the country with over 8.8 million doses of COVID-19 as of June 2022.

## Conclusion, Implications, and Limitations

The wave of the COVID-19 pandemic threatened human health and National Security. Among the devastation brought in the wake of the COVID-19 pandemic is the disruption of economies and widening poverty gaps across the globe. There has been a loss of employment and livelihood, and people suffer from anxiety due to social contacts’ loss. However, Tanzania’s coverage of COVID-19 vaccination remains significantly lower compared with other nations. This is due to the delay in introducing the COVID-19 vaccines into the country. People’s perception towards vaccines is still poor due to a lack of trust in the vaccines as well as misinformation and false news about the efficacy of the vaccine in some media. There is a strong need for advocacy with high-level political, community, and religious leaders and increased access to COVID-19 vaccination. In addition, a mass campaign to scale up vaccination activities with adequate resource mobilization is needed. This means that cooperation between the government and other stakeholders such as NGOs is vital to create awareness in the community on the need to accept the vaccine in order to curtail the spread of the virus as well as see to the end of the pandemic.

This study examined how the Tanzanian government, in collaboration with other stakeholders, is responding to the pandemic to improve global health security and community well-being. The study’s data was obtained from secondary sources including government communication, as well as international organizations like the WHO. The limitation of this study has to do with the data sources used. It would be more appropriate if primary data was used to get first-hand information about the issue which gives more credence to the findings as the data from primary sources seems more credible than from secondary sources which are likely to be massaged to achieve certain objectives. Future studies could therefore extend this research by using other sources of data including interviews and questionnaires to gauge citizens’ understanding of the issue at stake.

## Data Availability

All data generated or analyzed during this study are included in this published article.

## References

[1] World Health Organization (2020). Coronavirus disease 2019 (COVID-19): Situation Report-128.

[2] Stoeva P (2020). Dimensions of Health Security-A Conceptual Analysis. Global Challenges (Hoboken, NJ).

[3] Aldis W (2008). Health security as a public health concept: a critical analysis. Health Policy Plan.

[4] Farran S, Smith R. The ‘Pacific way’ of responding to the COVID-19 pandemic. The Round Table. 2021;110(2):217–31. 10.1080/00358533.2021.1904593.

[5] Hassan HA (2021). The securitisation of COVID-19 in Africa: Socio-economic and political implications. Afr Security Rev.

[6] Kosaka M, Hashimoto T, Ozaki A, Tanimoto T, Kami M (2021). Delayed COVID-19 vaccine roll-out in Japan. Lancet.

[7] Roberts SL, Kelman I (2022). Global health security and islands as seen through COVID-19 and vaccination. Global Public Health.

[8] McKinsey Insights (2022). A data-driven approach to addressing COVID-19 vaccine uptake in Africa, August 2, 2022. https://www.mckinsey.com/industries/healthcare-systems-and-services/our-insights/a-data-driven-approach-to-addressing-covid-19-vaccine-uptake-in-africa

[9] Igwe PA, Ochinanwata C, Ochinanwata N, Adeyeye JO, Ikpor IM, Nwakpu SE, Egbo OP, Onyishi IE, Vincent O, Nwekpa KC, Nwakpu KO, Adeoye AA, Odika PO, Fakah H, Ogunnaike OO, Umemezia EI (2020). Solidarity and social behaviour: how did this help communities to manage COVID-19 pandemic?. Int J Sociol Soc Policy.

[10] GHSA (2019). Global Health Security Index, 2019 Report.

[11] International Health Regulations (IHR) (2005).

[12] Nwajiuba CA, Igwe PA, Akinsola-Obatolu AD, Ituma A, Binuomote MO (2020). What can be done to improve higher education quality and graduate employability in Nigeria? A stakeholder approach. Industry High Educ.

[13] Schreier M, Flick U (2014). Qualitative content analysis. The SAGE Handbook of Qualitative Data Analysis.

[14] World Health Organization (2021). AFRO Report (2020-2021).

[15] Mutambo A (2021). Third Wave of Covid-19 Hits A Battered Kenyan Economy.

[16] World Health Organization (2019). Considerations in adjusting public health and social measures in the context of COVID-19: Interim guidance.

[17] Faria, J., (2022). Statistics research expert for Africa, published March 2022

[18] The United Republic of Tanzania, Coronavirus Statistics 2nd September 2022.

[19] Lo Yuk-ping C, Thomas N, Yuk-Ping L (2010). How is health a security issue? Politics, responses and issues. Health Policy Plan.

[20] USAID (2020). USAID Fact Sheet on Covid-19 Assistance in Tanzania.

[21] National Action Plan for Health Security (2017-2021). United Republic of Tanzania

[22] World Health Organisation [WHO]. World Health Report 2020; Health Systems; Improving Performance. Geneva: WHO; 2020. https://apps.who.int/iris/handle/10665/42281.

[23] Salunke S, Dharmesh Kumar L (2017). Multisector approach for promoting public health. India J Public Health.

[24] The United Republic of Tanzania, Ministry of Education science and Technology April 2020; Tanzania Basic Education sector response and Recovery Plan Due to effect of COVID-19.

[25] Ministry of Finance and Planning, Implementation of the Tanzania Covid-19 Socioeconomic Response and Recovery Plan (TCRP), Second Quarter Progress Report (January-March 2022).

[26] Patterson SA (2022). The Tanzanian state response to COVID-19: Why low capacity, discursive legitimacy, and twilight authority matter. WIDER Working Paper 2022/34, United Nations University.

[27] Mfinanga S, Mnyambwa N, Minja D, Ntinginya NE, Ngadaya E, Makani J, Makubi A (2021). Tanzania’s Position on the COVID-19 Pandemic. Lancet.

[28] Global Fund (2021). The Impact of Covid-19 on HIV, TB and Malaria Services and Systems for Health: A Snapshot from 502 Health Facilities Across Africa and Asia.

